# Infectious Complications in Paediatric Haematopoetic Cell Transplantation for Acute Lymphoblastic Leukemia: Current Status

**DOI:** 10.3389/fped.2021.782530

**Published:** 2022-02-10

**Authors:** Olga Zajac-Spychala, Stefanie Kampmeier, Thomas Lehrnbecher, Andreas H. Groll

**Affiliations:** ^1^Department of Pediatric Oncology, Hematology and Transplantology, Poznan University of Medical Sciences, Poznań, Poland; ^2^Institute of Hygiene, University Hospital Münster, Münster, Germany; ^3^Division of Pediatric Hematology and Oncology, Hospital for Children and Adolescents, University Hospital, Johann Wolfgang Goethe-University, Frankfurt, Germany; ^4^Infectious Disease Research Program, Center for Bone Marrow Transplantation and Department of Pediatric Hematology/Oncology, University Children's Hospital Münster, Münster, Germany

**Keywords:** acute lymphoblastic leukaemia, haematopoietic stem cell transplantation, infection, bacteria, virus, fungus

## Abstract

Haematopoietic stem cell transplantation (HSCT) in paediatric patients with acute lymphoblastic leukaemia (ALL) is associated with a variety of infectious complications which result in significant morbidity and mortality. These patients are profoundly immunocompromised, and immune reconstitution after HSCT generally occurs in astrictly defined order. During the early phase after HSCT until engraftment, patients are at risk of infections due to presence of neutropenia and mucosal damage, with Gramme-positive and Gramme-negative bacteria and fungi being the predominant pathogens. After neutrophil recovery, the profound impairment of cell-mediated immunity and use of glucocorticosteroids for control of graft-vs.-host disease (GvHD) increases the risk of invasive mould infection and infection or reactivation of various viruses, such as cytomegalovirus, varicella zoster virus, Epstein-Barr virus and human adenovirus. In the late phase, characterised by impaired cellular and humoral immunity, particularly in conjunction with chronic GvHD, invasive infections with encapsulated bacterial infections are observed in addition to fungal and viral infections. HSCT also causes a loss of pretransplant naturally acquired and vaccine-acquired immunity; therefore, complete reimmunization is necessary to maintain long-term health in these patients. During the last two decades, major advances have been made in our understanding of and in the control of infectious complications associated with HSCT. In this article, we review current recommendations for the diagnosis, prophylaxis and treatment of infectious complications following HSCT for ALL in childhood.

## Introduction

Allogeneic haematopoietic stem cell transplantation (allo-HSCT) is needed to cure a subpopulation of children with *de novo* and relapsed acute lymphoblastic leukaemia (ALL). However, allo-HSCT is associated with significant transplant-related mortality, ranging from 5 to 24%, due to serious infections or acute or chronic graft-vs.-host disease (GvHD), while secondary malignancies, organ dysfunction and compromised quality of life may pose additional problems (1–3). Despite advances in the HSCT procedure and refinements in supportive care strategies over the last 20 years, infections remain an important cause of morbidity and mortality after HSCT (4).

## Risk Factors for Infectious Complications

A greater depth and longer duration of myelosuppression and immunosuppression increases the risk that ALL patients will develop an infection that will take a more severe and complicated course. Patients with expected neutropenia <500/μL for at least 8 days are regarded to be at high risk of developing an infection with a complicated course (5, 6). While it is generally presumed that patients after allo-HSCT are amongst those with neutropenia lasting for 8 days or longer, all are at high risk of complicated infection. In addition to the presence of indwelling central venous catheters (CVCs), a risk factor for severe infectious complications in paediatric patients undergoing allo-HSCT for ALL is delayed immune reconstitution (7, 8).

The risk of infectious complications and the type of pathogen varies according to the timing after HSCT, and pre-transplant, transplant and post-transplant factors contribute to this risk. Infections after HSCT may derive from a patient's microbial flora, be a reactivated latent infection, or be a primary infection, with the latter being a common situation in children (9). Assessing each patient's pretransplant infectious disease status is an important part of the HSCT procedure, allowing additional therapy prior to HSCT to be applied if required and/or to identify possible latent infections that may reactivate early in the post-transplant period. Moreover, careful assessment of each patient's history of pretransplant infection and colonisation is necessary to guide secondary antimicrobial prophylaxis and/or treatment if the patient develops neutropenic fever in the early phase after HSCT (10, 11).

The post-transplant period is traditionally divided into three phases: (1) the pre-engraftment phase (the period up to neutrophil engraftment, which is defined as an absolute neutrophil count of >500 cells/mL on three consecutive days); (2) the post-engraftment phase (from neutrophil engraftment until day 100); and (3) the late phase (day >100) (9).

**Figure 1:**
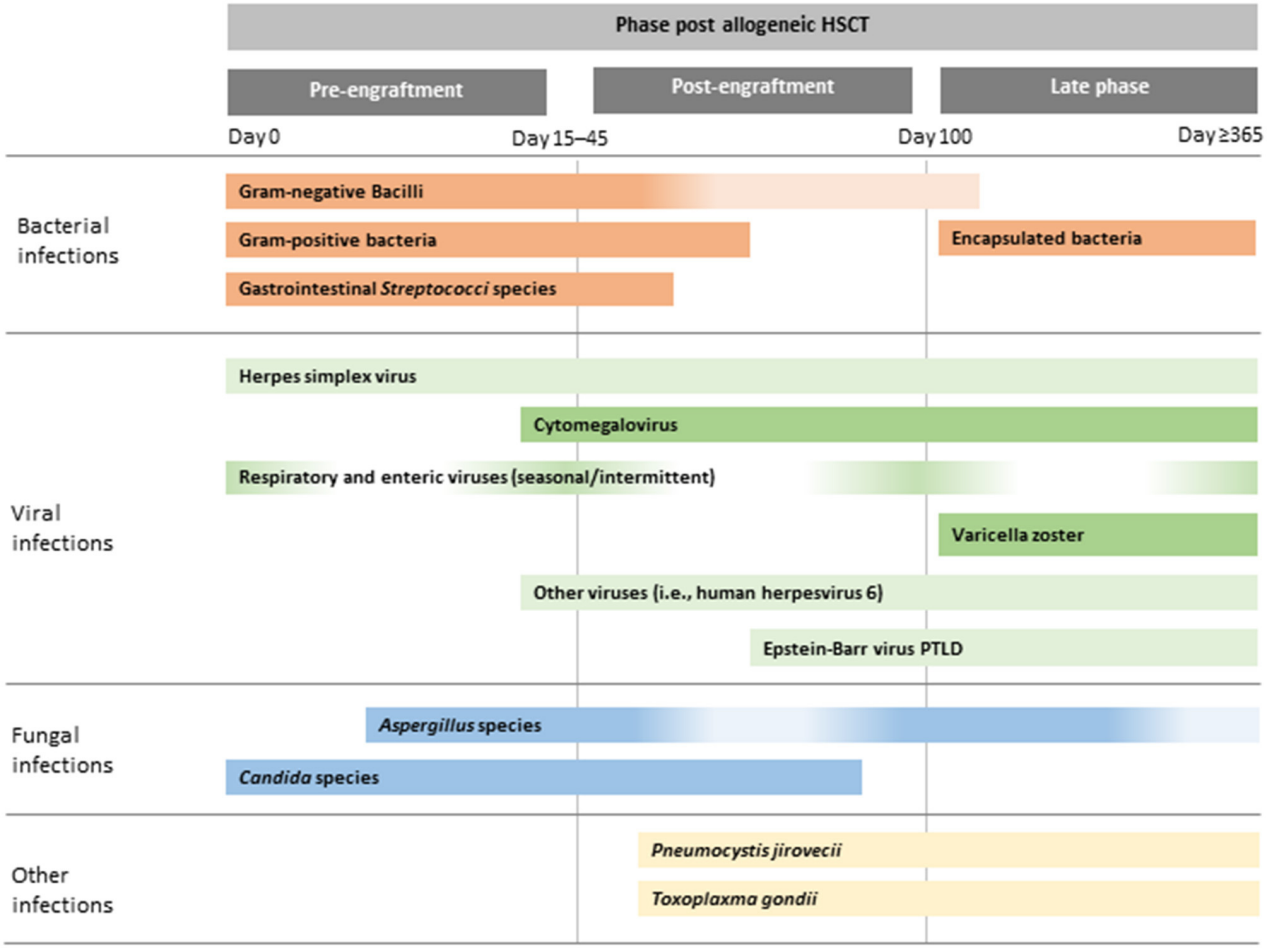
Illustrative chronology of infectious complications after allogeneic HSCT. Greater depth of colour indicates more common infections. PTLD, post-transplant lymphoproliferative disease; HSCT, haematopoietic stem cell transplantation.

In the pre-engraftment phase, infections are generally related to complications of prolonged and severe neutropenia and disruption to the normal host immune barriers (e.g., presence of mucositis and indwelling catheters). Bloodstream infections (BSI) occur most frequently during this time, although incidence rates and epidemiology in paediatric HSCT vary widely by institution, geographic location, centre and underlying HSCT factors (12, 13). After neutrophil engraftment, BSI may also occur, especially in children with renal or hepatic dysfunction and the presence of GvHD (14). In the post-engraftment period, infections are primarily related to ongoing profound defects in cellular immunity from the conditioning regimen and prophylaxis and/or treatment of GvHD. During this period, the reactivation of viruses, especially cytomegalovirus (CMV) and human adenovirus (hAdV) in haploidentival transplants predominates (15). Infections in the late phase are rare in HSCT recipients in ALL remission without GvHD (Figure 1). However, the risk of and severity of infections during this time period are directly related to GvHD and its immunosuppressive treatment. Immune defects associated with GvHD include those related to humoral and cellular immunity and functional hyposplenism. Thus, patients with GvHD are at greater risk of infections with viruses, filamentous fungi and encapsulated bacteria. In addition, steroid-refractory GvHD is treated with multiple immunosuppressive agents with distinct immune targets, further altering the risk of and clinical manifestations of infections (16, 17).

Although the risk of infections caused by bacteria, viruses and fungi may be different during certain timepoints after HSCT, each infectious complication may occur at any time until successful immune reconstitution (9).

## The Principles of Diagnosis of Infectious Complications

Diagnostic procedures for infectious complications can be divided into those performed before the onset of fever or other signs and symptoms of infections as routine screening and those performed in the event of fever or signs and symptoms of infection.

### Screening

Routine surveillance using blood cultures in the absence of fever or other signs of infection are discouraged in allo-HSCT recipients (12, 13). Likewise, general screening for invasive aspergillosis by serial determination of galactomannan antigen or 1,3-β-D-glucan is not recommended by the European Society of Bone and Marrow Transplantation (EBMT) and European School of Haematology in patients who are receiving mould-active prophylaxis (18). In individual patients at increased risk of invasive infection with *Aspergillus* spp. who are in the deep neutropenic phase (e.g., pre-engraftment), twice-weekly galactomannan and/or 1,3-β-D-glucan surveillance may be considered (19, 20).

In all paediatric patients after allo-HSCT, the regular monitoring for CMV, Epstein-Barr virus (EBV) and hADV DNA should be routinely performed up to 60 days after transplantation. Monitoring for hAdV should be continued in haploidentical SCT until T cell recovery is observed. Monitoring for CMV and EBV should be prolonged for up to 180 days, when an unrelated donor was used and/or when GvHD is present (21). Screening for other viruses, including adenovirus, herpes simplex virus type 1 (HSV-1) or human herpesvirus 6 (HHV-6) is recommended by multiple guidelines in patients displaying additional risk factors for each type of viral infection (22–24). The routine use of polymerase chain reaction (PCR) quantification of viral load makes it possible to detect viraemia earlier (25).

In all HSCT recipients, especially those with fever or infection, regular thorough physical examination is mandatory and cannot be replaced by any laboratory test.

### Diagnostic Tests in Those With Signs and Symptoms

It is recommended by multiple experts to obtain blood cultures from all CVC lumens and to consider also peripheral blood cultures in the event of fever or other signs or symptoms of infection (26–28). In BSI caused by *Staphylococcus aureus* or *Candida* spp., CVCs should be removed whenever possible, independent of the exact source of infection (14).

In the presence of fever or suspected infection, cultures of blood, urine and other specimens from possible sources of infection, PCR studies, blood gas analysis, biochemical analyses including C-reactive protein (CRP) and procalcitonin, as well as various imaging techniques should be ordered immediately with consideration of the most probable infectious agents to which the patient might have been exposed, medical history and previously encountered pathogens during past treatments (21). Chest X-rays are commonly discouraged to diagnose lung infection in cancer patients, since infiltrates are frequently invisible (29, 30). High-resolution thoracic computed tomography (CT) scanning without contrast enhancement has a significantly higher sensitivity than chest X-ray and is recommended in patients with respiratory symptoms or persisting fever despite antimicrobial treatment for 72–96 h (5, 28, 31). Moreover, it is suggested to consider imaging of abdomen in patients without localised signs or symptoms because studies have identified cases of imaging consistent with invasive fungal diseases (IFD) in patients without localised signs or symptoms (weak recommendation, low-quality evidence) (32, 33). The ideal imaging modality is not known, but ultrasound is readily available, is not associated with radiation exposure and usually does not require sedation; thus, ultrasound is likely to be preferable over CT or magnetic resonance imaging for abdominal assessment (28). Diagnostic bronchoscopy or bronchial or bronchoalveolar lavage for patients with pulmonary infiltrates should be applied whenever possible. Further diagnostics (e.g., abdominal or central nervous system [CNS] imaging) might also be required, depending on symptoms, clinical signs and laboratory parameters (5). However, simultaneously to intensive diagnostic procedures, antibiotic treatment should be administered immediately to all patients with signs and symptoms of a bacterial infection in the early post-transplant period.

Early diagnosis is also key to the successful management of IFDs. Standard procedures encompass blood cultures for yeast and some of the rare moulds; cultures and microscopic examination of appropriate specimens; and imaging studies as determined by clinical findings. In the recently updated ECIL-8 guidelines, a CT scan of the lungs is strongly recommended in patients with febrile granulocytopaenia that persists beyond 96 h or with focal clinical findings; since unspecific radiographic findings are common, typical and non-typical pulmonary infiltrates should prompt further diagnostic work-up and initiation of mould-active antifungal treatment. Of note, due to the high frequency of not always symptomatic CNS involvement (34), appropriate cranial imaging should be considered in all patients with probable or proven pulmonary mould infection (B-II) (35). Galactomannan testing of serum is strongly recommended in granulocytopaenic patients with prolonged or new fever and in patients with abnormalities in pulmonary CT imaging. Whenever specimens are obtained for diagnostic work-up for pulmonary or cerebral IFDs, galactomannan testing of bronchoalveolar lavage (BAL) and cerebrospinal fluid (CSF) is recommended; molecular methods for detection of fungal nucleic acids in BAL, CSF, aspirates and tissues are also recommended, preferentially in a national fungal reference laboratory. (35). However, it should be also emphasised that especially in small children some diagnostic procedures are more difficult to perform.

### Prevention of Infections

Approaches to prevent infections are based on a careful risk-benefit assessment and include the general infection control measures of contact precautions and microbiological surveillance of both the patient and hospital environment, regular thorough physical examination, antimicrobial chemoprophylaxis, administration of immunoglobulins in hypogammaglobulinaemic patients, and post-transplant vaccinations.

### General Precautions

To identify patients at risk of certain infectious diseases and to prevent transmissions of multidrug resistant (MDR) or highly virulent organisms, comprehensive screening is recommended prior to and during transplantation. Screening procedures should assess for colonisation with methicillin-resistant *S. aureus*, vancomycin-resistant Enterococci and MDR Gramme-negative bacteria. Additionally, appropriate tests for relevant viral diseases (e.g., adenovirus viraemia) that are particularly highly transmissible, CMV, EBV and toxoplasmosis are recommended to avoid their nosocomial dissemination or to initiate pre-emptive treatment (36, 37). In the event of the detection of a highly transmissible microorganism contact precautions are a prerequisite to exclude cross-patient transfer (37–39). Healthcare workers with transmissible diseases should not work in direct patient care and ideally should stay at home to prevent the nosocomial spread of their disease (37, 39).

All transplanted patients should be housed in a single protective environment room equipped with >12 air exchanges per hour, high efficiency particulate air (HEPA) filters, directed air flow and positive air pressure differential (Pa) between the room and the hallway of >2.5 Pa (10, 37–39) to maintain a low count of environmental spores. Apart from environmental surfaces, special attention has to be placed on the construction and hygienic maintenance of sanitary and water supply systems as they may serve as a source of biofilm-producing and other MDR organisms or *Legionella* spp (37, 40).

The dietary needs of paediatric patients after allo-HSCT are an important issue with little evidence-based foundation; as expressed elsewhere, general guidance such as a “cook it, peal it, or forget it” approach for selection of food items (37) and a “clean, separate, cook and chill” approach for preparing food items (39) is easy to understand and to follow and may serve as basis for dietary recommendations. Sources of infectious agents after discharge to the outpatient setting might include water, dust, plants and flowers, decaying biological waste, certain food items, pets and contact with other individuals. The findings of a recent study in children with acute myeloblastic leukaemia suggest that a strict neutropenic diet and strict policies regarding restriction of social contacts (e.g., school attendance) and restriction of pets at home do not decrease the rate of infections (41). In the absence of strong evidence, appropriate measures include attention to the cleanliness of sanitary systems, kitchen appliances and surfaces; the avoidance of carpets, flowers and plants in the house; avoidance of close contact to biological waste or hygienic interactions with pets; frequent hand disinfection and attention to personal hygiene; keeping distance from social contacts; wearing masks where appropriate; and avoidance of raw-meat products and unpasteurized milk products. Detailed recommendations that consider the dynamics of the net state of immunosuppression post-transplant including immune recovery (CD4^+^ T cell and granulocyte count), presence of GvHD, level of immunosuppression and infection rates have been elaborated by the Paediatric Diseases Working Party (PDWP) of the EBMT and can be found elsewhere (39).

## Chemoprophylaxis

### Antibacterial Prophylaxis

In principle, antibacterial chemoprophylaxis including for Gramme-positive and Gramme-negative organisms is a valid consideration to reduce invasive bacterial infections post HSCT but the potential for adverse effects and the emergence of resistance have to be carefully weighed against hard endpoints of efficacy, including reduction of invasive infections and infection-related and overall mortality (42). Corroborating paediatric-specific guidelines developed by an international panel (43) and in line with recommendations issued by the PDWP of the EBMT (39), the recently published recommendations from the 8th European Conference on Infections in Leukaemia (ECIL-8) do not recommend the routine use of antibacterial chemoprophylaxis in children undergoing HSCT during the pre-engraftment phase (recommendation against use, evidence level I) (42). The recommendation is mainly based on a large, prospective, randomised study which did not find that levofloxacin prophylaxis given from day−2 until engraftment significantly reduced mortality or the risk of BSI (44) as well as available clinical trials and meta-analyses in paediatric and adult patients indicating that antibacterial prophylaxis might possibly lead to increased resistance to fluoroquinolone and other important broad-spectrum beta-lactam antibiotics in colonising bacteria (42). This recommendation does not exclude the use of antibacterial prophylaxis in individual patients for whom the potential individual benefit exceeds potential negative consequences.

### Antifungal Prophylaxis

Primary antifungal prophylaxis is strongly advised in the pre-engraftment and post-engraftment phases until immune reconstitution and discontinuation of immunosuppression or in the context of augmented immunosuppression for GvHD to reduce disease-related morbidity and mortality in all transplanted patients (35, 39). Antifungal agents recommended for paediatric patients by the ECIL-8 group include fluconazole (; only if the institutional incidence of invasive mould infections is low, or if there are active diagnostic and therapeutic algorithms for mould infections; not to be used post engraftment in allo-HSCT where mould infections dominate), posaconazole, and, with lesser strength, itraconazole and voriconazole (35). Further options include liposomal amphotericin B, micafungin, and caspofungin (no grading). Drug–drug interactions and drug-associated adverse effects need to be considered on an individual basis (35, 45–47). These recommendations are based on efficacy data from Phase III clinical trials in adults, the existence of paediatric pharmacokinetic data and dosing recommendations, paediatric safety data and supportive efficacy data with consideration of regulatory approval for use of agents in paediatric patients (35, 45).

### *Pneumocystis jirovecii* Prophylaxis

*Pneumocystis jirovecii* pneumonia is a life-threatening disease in allo-HSCT recipients and adequate prophylaxis is critical. Trimethoprim/sulfamethoxazole is the preferred drug combination for primary prophylaxis; recommended dosing regimens in children include 150/750 mg/m^2^/day in one or two doses per day or the same dose on 2 or 3 days per week (48). Inferior second-line alternatives include aerosolized pentamidine (300 mg once per month in children >5 years) and dapsone, atovaquone or intravenous pentamidine (39, 48, 49). Prophylaxis is usually started after engraftment and continued during immunosuppressive therapy until protective immune recovery is achieved (39, 48).

### Antiviral Prophylaxis

Among CMV-seropositive HSCT recipients, approximately 80% develop CMV reactivation and 20–35% progress to CMV disease if no preventative steps are taken; mortality of established disease is up to 50% despite treatment. Because of the toxicities of ganciclovir, foscavir and cidofovir, pre-emptive therapy has been the preferred approach; nevertheless, pre-emptive therapy is started after CMV viraemia is detected and any level of viraemia is associated with an increased risk of overall mortality (39, 50–53). Letermovir is a new antiviral agent that inhibits CMV through a novel mechanism involving the viral terminase complex (54). It is available as an intravenous and oral formulation, has a favourable pharmacokinetic and safety profile, and has been approved on the basis of the results of a randomised, double-blind, placebo-controlled clinical trial in CMV-seropositive adult recipients of allo-HSCT for primary prophylaxis of CMV reactivation prior to engraftment (55). Paediatric development is under way and, pending paediatric approval, will fundamentally change the management of CMV-seropositive HSCT recipients.

The risk of reactivation of HSV and VZV in seropositive HSCT recipients at some point after transplantation is close to 80% for each virus. In consideration of the high morbidity and the potential for patient-to-patient transmission, antiviral drug prophylaxis with acyclovir, valaciclovir or famciclovir is strongly recommended for VZV-seropositive patients for 1 year or longer in the presence of GvHD and immunosuppressive therapy. For VZV-seronegative HSV-seropositive patients, the recommended duration of prophylaxis generally matches the duration of immunosuppression (36, 39, 56, 57). If breakthrough infection occurs, drug resistance should be considered and genotyping ordered to guide further treatment. Apart from foscarnet, options to target acyclovir-resistant isolates include agents that target the viral helicase-primase complex of HSV (pritelivir, amenamevir) and VZV (amenamevir). These agents are currently available for adults within a compassionate use program (pritelivir) or through international pharmacy (amenamevir is approved in Japan). Paediatric dosing recommendations are currently lacking (58).

## Administration of Immunoglobulins

In both the inpatient and outpatient setting, severe hypogammaglobulinaemia (e.g., immunoglobulin G <4 g/L) may be associated with an increased rate of infections and, despite the lack of strong evidence, international guidelines produced by multiple societies currently recommend immunoglobulin substitution in HSCT recipients with severe hypogammaglobulinaemia for the prevention of invasive bacterial and viral respiratory infections (10, 37).

### Management of Infectious Complications

The pre-engraftment phase after HSCT is complicated by mucosal damage and neutropenia; the severity and duration of these problems depend on the conditioning regimen given. In addition, a central venous line disrupts the skin barrier in most patients. Unfortunately, the management of CVC-related infections remains difficult, and there are still open questions such as whether catheters should be removed or not (59). All these factors significantly increase the risk of bacterial infections caused by Gramme-negative bacteria arising from the normal gastrointestinal flora and by Gramme-positive bacteria associated with indwelling catheters (Table 1) (60). Unfortunately, the prevalence of resistant bacterial pathogens has significantly increased over the last decade, which is a worldwide phenomenon (61). This is important because studies in adults with cancer who are infected with MDR Gramme-negative bacteria have demonstrated that these patients often receive inadequate empirical antibacterial therapy resulting in poorer outcome than Gramme-positive (62). Although a recent study in children undergoing HSCT for acute leukaemia reported low resistance rates for bacteria isoated from the stool (e.g., fluoroquinolone resistance 1%, cefepime 2.5%, imipenem 0%) (44) local resistance rates for colonisation and infection vary widely as reported in children undergoing therapy for cancer, which depends, at least in part, from antibacterial prophylaxis (63, 64). Therefore, regular local epidemiologic surveillance is critical and has an important impact on the choice of antibiotic compound used.

**Table 1:** Risk factors for bacterial infectious complications.

**Intention**	**Recommendation and grading**	**Comment**	**References**
Antibacterial prophylaxis	Whereas systemic antibacterial prophylaxis may be considered in children with AML and relapsed ALL receiving intensive chemotherapy, this recommendation is not given for neutropenic children undergoing HSCT (Weak recommendation; high-quality evidence)	Recommendations based on the results of a systematic review of randomised trials of systemic antibacterial prophylaxis (Egan Cancer Med 2019)	Lehrnbecher et al. (43)
	Routine antibacterial prophylaxis for paediatric patients with neutropenia during the pre-engraftment stage of HCT is not recommended (grade D recommendation, level of evidence III)	Recommendation by ECIL-8 based on data from randomised trials and meta-analyses, information from long-term observational studies on resistance	Lehrnbecher et al. (42)
Empirical antibacterial therapy	a. Use monotherapy with an antipseudomonal b-lactam, a fourth-generation cephalosporin, or a carbapenem as empirical therapy in paediatric high-risk FN (strong recommendation, high-quality evidence) b. Reserve the addition of a second gramme-negative agent or a glycopeptide for patients who are clinically unstable, when a resistant infection is suspected, or for centres with a high rate of resistant pathogens (strong recommendation, moderate-quality evidence).	International Paediatric Fever and Neutropenia Guideline Panel includes representation from paediatric oncology, infectious diseases, nursing, and pharmacy, as well as a patient advocate and a guideline methodologist from 10 different countries	Lehrnbecher et al. (28)
	Clinically stable patients at low risk of resistant infections: monotherapy with an antipseudomonal non-carbapenem β-lactam and β-lactamase inhibitor combination, or with fourth-generation cephalosporin (grade A recommendation, level of evidence IIr) Clinically unstable patients, even when at low risk of resistant infections: carbapenem, with or without a second anti-Gramme-negative agent, with or without a glycopeptide (grade A recommendation, level of evidence IIt). Patients who are colonised or were previously infected with resistant Gramme-negative bacteria, or in centres with a high rate of resistant pathogens: empirical treatment should be adjusted on the basis of the results of resistance testing (grade A recommendation, level of evidence IItu)	ECIL-8 recommendations	Lehrnbecher et al. (42)
Therapy of documented bacterial infection	If a causative pathogen is identified, the patient should be treated according to the causative organism identified (assuming it is a plausible pathogen). the choice of which should be guided by *in-vitro* susceptibility tests, including minimum inhibitory concentrations when available (recommendation 4, grade A, level of evidence IItu)	ECIL-8 recommendations	Lehrnbecher et al. (42)
Prophylaxis of fungal infections	Primary antifungal prophylaxis is strongly recommended for patients undergoing allogeneic HCT in the pre-engraftment and in the post-engraftment phase until immune reconstitution, or in situations of augmented immunosuppressive treatment in the context of graft-vs.-host disease (i.e., use of additional immunosuppressive interventions to control overt graftvs.- host disease, including, but not limited to, the use of glucocorticosteroids in therapeutic doses (≥0·3 mg/kg per day prednisone equivalent) or anti-inflammatory antibodies) (grade A recommendation, level of evidence: IIt).	ECIL-8 recommendations based on risk assessment in paediatric HCT patients and results of interventional studies in adults	Groll et al. (35)
	Administer systemic antifungal prophylaxis to children and adolescents undergoing allogeneic HSCT pre-engraftment and to those receiving systemic immunosuppression for the treatment of graft-vs. host (strong recommendation, moderate quality evidence).	Recommendations developed by an international multidisciplinary panel on the basis of a systematic review of systemic antifungal prophylaxis in children and adults with cancer and HSCT recipients.	Lehrnbecher et al. (43)
Empirical antifungal therapy	If empirical therapy is chosen as a strategy, it should be initiated in granulocytopenic patients after 96 h of fever of unclear cause that is unresponsive to broad-spectrum antibacterial agents (grade B recommendation, level of evidence: II)	ECIL-8 recommendation based on clinical trials in paediatric and adult patients.	Groll et al. (35)
	Initiate empirical antifungal therapy in patients with granulocytopenia and prolonged (≥ 96 h) fever unresponsive to broadspectrum antibacterial agents (strong recommendation, high-quality evidence).	Recommendations developed by an international multidisciplinary panel on the basis of a systematic review of empirical management of fever and neutropenia in	Lehrnbecher et al. (28)
		children and adults with cancer and HSCT recipients.	
Pre-emptive or diagnostic-driven antifungal therapy	If chosen as a strategy, rapid availability of pulmonary CT and galactomannan assay results is a prerequisite and capability of performing bronchoscopies with bronchoalveolar lavage is desirable. The sensitivity of galactomannan in serum might be lower in patients on mould-active prophylaxis (grade B recommendation, level of evidence: II)	ECIL-8 recommendation based on clinical trials in paediatric and adult patients.	Groll et al. (35)
Therapy of proven or probable fungal infections	Treatment of proven or probable invasive fungal infections include general magament principles including prompt initiation of antifungal therapy, resistance testing, source control and control of predisposing conditions. Echinocandins or liposomal amphotericin B are recommended for the first-line treatment of invasive Candida spp infections before species identification (grade A recommendation, level of evidence: IIt) and intravenous voriconazole (grade A recommendation, level of evidence: IIt)or liposomal amphotericin B (grade B recommendation, level of evidence: IIt) for invasive Aspergillus infections.	ECIL-8 recommendation based on clinical trials in paediatric and adult patients.	Groll et al. (35)

*(adapted from Lehrnbecher et al. J Pediatr Hematol Oncol 1997: 19;399–417)*.

It is the longstanding standard of care to start empirical antibacterial therapy in neutropenic children at the onset of fever or at any other sign or symptom of possible infection (28, 65). As a systematic review on empirical therapy in neutropenic paediatric HSCT recipients with fever found that aminoglycoside-containing combination therapy did not decrease treatment failures and mortality compared to guideline-consistent monotherapy (66), the ECIL-8 group strongly recommends an antipseudomonal non-carbapenem beta-lactam plus beta-lactamase inhibitor or monotherapy with a fourth-generation cephalosporin for clinically stable patients at low risk of resistant infections (42). In clinically unstable patients, a carbapenem with or without a second anti-Gramme-negative agent and/or glycopeptide is strongly recommended, whereas in patients who are colonised or had a previous infection with resistant Gramme-negative bacteria or in institutions with a high rate of resistant pathogens, empirical treatment should be adjusted based on results of resistance testing.

When a causative pathogen has been identified, there is a strong recommendation to narrow the empirical antibiotic regimen and to adapt it to this organism and to the results of *in vitro* susceptibility tests (42). In those patients who are colonised or had a previous infection with resistant pathogens, de-escalation after 72–96 h of initial empirical therapy should be strongly considered. In this respect, any aminoglycoside, fluoroquinolone, colistin or antibiotic directed against resistant Gramme-positive pathogens should be discontinued if given in combination and initial carbapenem therapy should be changed to a narrower-spectrum antibiotic. It is less clear whether in individual HSCT recipients with fever of unknown origin (i.e., without clinically or microbiologically documented infection), empirical intravenous antibiotics can be discontinued after a minimum of 72 h of therapy, even prior to signs of haematological recovery, if the patient has always been haemodynamically stable and has been afebrile for 24–48 h (42). Therefore, assessment of the safety and efficacy of early step-down strategies is a future goal of clinical trials, which might be facilitated by new serum biomarkers as diagnostic and monitoring tools. In addition, in view of the emerging resistance of bacterial pathogens, new antibiotics are urgently needed and must be used prudently.

### Management of Viral Infections

The most common viral infections in the paediatric recipients of allo-HSCT belong to the Herpesvirus family. The majority of herpes virus infections after transplantation result from reactivation of latent virus. CMV, HSV and VZV account for most disease caused by the Herpesvirus family, although there has been increasing recognition of HHV-6 in this setting (67). EBV reactivation after HSCT can lead to clonal proliferation of CD20^+^ B cells, potentially causing EBV-related post-transplant lymphoproliferative disease (PTLD) which has become an increasingly common management problem in allo-HSCT recipients. Haemorrhagic cystitis due to human adenovirus or BK virus is a painful disease that is difficult to treat; alongside systemic adenovirus infection it can hamper the outcome of HSCT (68). Other potential causes of life-threatening infectious complications in allo-HSCT recipients are respiratory pathogens including influenza, parainfluenza, respiratory syncytial virus (RSV) and the recently recognised new member of the Paramyxoviridae family human metapneumovirus (69).

Infections due to human adenovirus, influenza, RSV, parainfluenza virus type 3 and other respiratory viruses are encountered in all phases after allo-HSCT, including the pre-engraftment, post-engraftment and late phases. Infections due to HSV are mostly seen during the pre-engraftment phase, whereas infections due to CMV and HHV-6 are seen in the early post-engraftment phase (<3 months) and EBV and VZV infections often occur after day 100 (late phase) (21).

Pre-emptive therapy for viral infections currently applied in allo-HSCT recipients aims to treat subclinical viral reactivation before clinical manifestations appear because during the immunocompromised state of transplanted patients there is insufficient host immunity to control viral replication. The first-line approaches to viral infections comprise tapering of immunosuppression and use of antiviral drug therapy. However, patients may not respond because of a lack of immune reconstitution, viral drug resistance or drug toxicity. Patients receiving serotherapy as part of conditioning (to deplete T cells) or glucocorticosteroids for control of GvHD are at higher risk of viral reactivation (68). Thus, routine monitoring of viral reactivation in the post-transplant setting usually includes molecular detection of viral DNA of the three most frequent viruses responsible for refractory infections, namely CMV, EBV andhAdV (70). Data on the incidence of viral reactivation, viral disease, standard treatment and rate of response are summarised in Table 2.

**Table 2:** Incidence of adenovirus, CMV and EBV reactivation, disease, pharmacological treatment and rate of treatment response in children after allogeneic HSCT.

**Virus**	**Viremia incidence**	**Viral disease incidence**	**Pharmacological treatment**	**Response rate**
Human adenovirus	15–30%	6–11%	Cidofovir, brincidofovir	60–80%
CMV	15–20%	4%	Ganciclovir, foscarnet, valganciclovir	70–80%
EBV	11%	1–7%	Rituximab	60–70%

*CMV, cytomegalovirus; EBV, Epstein-Barr virus; HSCT, haematopoietic stem cell transplantation. Adapted from Ottoviano et al. (68)*.

CMV infection, defined as the development of CMV viraemia, remains one of the most important viral infections after allo-HSCT, occurring in 15–20% children. Infection is usually the result of reactivation of endogenous virus, occurring in up to 80% of seropositive individuals. Seronegative individuals have a 30–40% chance of becoming infected when receiving unscreened blood products or stem cells from a seropositive donor (71). Two strategies are equally effective at preventing CMV infection after HSCT: (1) universal primary CMV antiviral prophylaxis given from the time of engraftment to day 100; or (2) viral surveillance with pre-emptive antiviral therapy when necessary (9).

Patients who have a reactivation of latent virus or become infected with CMV from an exogenous source may remain asymptomatic or develop clinical presentation with fever, bone marrow suppression and other organ involvement (with pulmonary involvement being the most common) (68). Other, rare localizations of CMV reactivation include gastrointestinal disease, hepatitis, encephalitis and retinitis, the latter if which was previously felt to be rare in allo-HSCT recipients (71).

Several drugs can be used to treatment CMV reactivation. The standard therapy is ganciclovir, although associated myelotoxicity precludes its useas standard preemptive therapy for CMV infection. Foscarnet is generally the next alternative to ganciclovir for CMV infections at this stage, although it is associated with a significant risk of renal toxicity. There are some early data on the use of oral valganciclovir in the bone marrow transplant setting, but myelotoxicity may still be a problem (68, 72, 73).

Further development of cellular therapies for viral infections focused on the specificities of T cells for different viruses, aiming to achieve higher response rates (74, 75). The first and most widely used protocols to develop virus-specific T cells were based on *in vitro* generation and expansion of T cells, leading to a final product comprising polyclonal T cells (recognising different immunogenic viral antigens). One of the main advantages of the *ex vivo* differentiation of virus-specific T cells is that it could overcome the potential obstacle represented by paucity of specific immunity for the virus in the donor immune system (68, 74). A novel and promsing approach may be the adoptive transfer of donor-derived T lymphocytes expressing an inducible human caspase 9 that may provide a robust immunologic benefit with immediate and sustained protection from major viral pathogens (76, 77).

After neutrophil engraftment, the absence of CD4-positive T-cell reconstitution predicts reactivation of viruses such as hAdV and EBV. Incidence of hAdV infection raises up to 30% being higher in children than in adult recipients (67) and its clinical manifestation varies from asymptomatic viremia to invasive localised and disseminated disease with mortality rate up to 80% (21). The most common transmission modalities are inhalation of aerosol droplets, direct conjunctival inoculation, faecal-oral route or contact with infected tissues or surfaces. Clinical disease syndromes associated with HAdV infections occur after primary infection or from reactivation of latent viruses. The optimal therapeutic strategy is unknown, although intravenous cidofovir may be used in patients with risk factors for disseminated hAdV disease. The outcome is usually hampered by T-cell lymphocytopenia and renal toxicity (74). Brincidofovir, a lipid conjugate of cidofovir, provided higher intracellular levels of active drugs and thus reduced adenoviral load more rapidly than cidofovir, however, due to organ toxicity, mainly related to the gastrointestinal tract is no longer in clinical development. Thus, within paediatric HSCT recipients, who apparently carry the greatest risk of severe and life-threatening infection courses, preemptive treatment based on virus detection prior to clinical manifestation should be applied.

Endogenous reactivation or graft-originated contamination may cause EBV-related disease among allo-HSCT recipients, and the most significant clinical syndrome is PTLD irrespective of acquisition route. Primary EBV infection, splenectomy, transplantation from a seropositive donor to a seronegative recipient, use of an unrelated and/or mismatched graft, use of T-cell depletion and anti-thymocyte globulin (ATG) are risk factors for PTLD (18). Thus, monitoring of EBV viral load in paediatric allo-HSCT recipients at high risk of PTLD is strongly recommended until the immunosuppressive therapy completed. The increased use of anti-CD20 monoclonal antibody (rituximab) has significantly reduced the incidence and mortality of EBV-driven PTLD in children; however, although such therapy can lead to excellent response rates when used as a pre-emptive strategy, efficacy as treatment of PTLD is around 60% (23, 68).

Infections due to HHV-6 may lead to engraftment delays or graft failure after paediatric allo-HSCT. They may also cause clinically relevant disease with a facial rash, occasional severe organ failure (lung, liver or CNS)—which is sometimes confused with encephalitis or acute GvHD—and, rarely, a fatal outcome in HSCT recipients. Viral reactivation needs to be distinguished from chromosomal integration. However, the exact prevalence of HHV-6 reactivation is not well documented since it is not part of routine viral monitoring in transplanted patients, and HHV-6 reactivation may be found in the absence of any associated clinical features (78). For now, there is no consensus on therapeutic, prophylactic or preventive strategies for HHV-6 infection/reactivation; however, ganciclovir, foscarnet or cidofovir are reported to be used in cases of HHV-6 reactivation (18).

### Management of Fungal Infections

While the precise incidence of IFDs following allo-HSCT is difficult to assess because of the almost universal use of antifungal prophylaxis and variable stringency in performing diagnostic procedures, incidence rates of around 10% are consistently observed with case fatality rates ranging from 20 to 70% and the poorest outcomes observed in disseminated disease, CNS involvement or persistent granulocytopenia (35, 79, 80). In a more recent systematic literature review of paediatric studies published between 1980 and 2016, a number of factors commonly associated with an increased risk of IFD were confirmed including prolonged granulocytopaenia, high-dose steroid exposure, and acute and chronic GvHD (81). Additional risk factors observed in several case series included increasing age (without a precise threshold), *a priori* determined transplant-related mortality risk >20%, admission to the intensive care unit, late or no lymphocyte engraftment, and, limited to invasive candidiasis, the presence of a CVC (35, 81).

*Candida* and *Aspergillus* spp. account for the majority of proven and probable IFDs with variable relative distribution in different series, institutions and countries (35, 79, 80, 82). The spectrum of invasive candidiasis in children closely resembles that seen in adults, with a predominance of catheter-associated candidaemia (80). *C. albicans, C. parapsilosis* and *C. tropicalis* are the most frequent species isolated; *C. auris* is an emerging pathogen that is notable for its nosocomial spread and potential resistance to more than one class of antifungal agents (83). Dissemination is observed in 10–20% of paediatric patients with candidaemia, while severe sepsis and/or septic shock occur in approximately 30% (80, 84–86).

Similar to adults, most paediatric patients with invasive aspergillosis present with pulmonary aspergillosis; dissemination to other sites, particularly the CNS, occurs in approximately 30% of cases (82, 87). *A. fumigatus* is most common cause, followed by *A. flavus* and *A. terreus*, although local differences may exist. Azole resistance is emerging and needs to be considered specifically in *A. fumigatus* infection (86). IFDs caused by non-Aspergillus moulds (i.e., *Fusarium* spp., *Scedosporium* spp., the agents of mucormycosis and others) present similarly to invasive aspergillosis but some of them may cause fungaemia and are more frequently associated with extrapulmonary forms of disease. The incidence of IFD caused by non-Aspergillus moulds is variable and accounts for 0–35% of all proven/probable fungal infections (82, 86–89). Intrinsic resistance of non-Aspergillus moulds to antifungal agents is frequent, and mortality appears to exceed 50% for most of the non-Aspergillus moulds. Of note, new guidelines have been published on mucormycosis, rare moulds and rare yeasts; these include paediatric-specific recommendations (90–92).

Empirical antifungal treatment is a well-established approach for persistently febrile granulocytopenic patients at high risk of IFD. If chosen as a strategy, empirical therapy should be initiated in granulocytopenic patients after 96 h of fever of unclear aetiology that is unresponsive to broad-spectrum antibacterial agents and should be continued until resolution of fever and granulocytopenia in the absence of suspected or documented IFD. A similar approach can be chosen in those granulocytopenic patients who develop recurrent fever after defervescence upon the initiation of broad-spectrum antibacterial agents (35). Based on the results of a recently completed multicentre, randomised clinical study comparing empirical vs. pre-emptive antifungal therapy (93), pre-emptive or diagnostically driven therapy is now recommended as an alternative strategy to empirical therapy with the prerequisite of rapid availability of pulmonary CT imaging and of galactomannan test results (35).

General management principles for IFDs are well established and include the prompt initiation of appropriate antifungal therapy, identification and resistance testing of all invasive isolates, source control, and management of predisposing conditions, as feasible (94). Echinocandins or liposomal amphotericin B (95–97) are strongly recommended for the first-line treatment of invasive *Candida* infections before species identification, and voriconazole and liposomal amphotericin B for first-line treatment of invasive aspergillosis (98, 99). Recommendations for mucormycosis, rare moulds and rare yeasts are beyond the scope of this article but have been updated recently by the European Confederation of Medical Mycology (ECMM) consortium (90–92) (Table 3).

**Table 3:** Summary of ECIL-8 recommendations for empirical, pre-emptive, and targeted therapy of IFDs (modified from Groll et al. ECIL-8 recommendations; for management of mucormycosis, rare moulds and rare yeast, please see the updated recommendations of the European Confederation of Medical Mycology (ECMM) consortium (91, 92).

**Recommendation and grading**	
**Empirical and pre-emptive antifungal therapy**	
If chosen as a strategy, empirical therapy should be initiated in granulocytopenic patients after 96 h of fever of unclear aetiology that is unresponsive to broad spectrum antibacterial agents	
	Options approved for this indication include caspofungin or liposomal amphotericin
*Pre-*emptive or diagnostically driven therapy is recommended as an alternative strategy to empirical therapy but requires rapid availability of pulmonary CT imaging and of galactomannan test results	
**Targeted therapy of invasive Candida infections**	
Echinocandins or liposomal amphotericin B are strongly recommended for first line therapy before species identification;	
	Voriconazole and fluconazole are secondary alternatives in this situation
**Targeted therapy of invasive Aspergillus infections**	
Recommendations for first-line therapy of invasive aspergillosis include voriconazole and liposomal amphotericin B	
	The combination of voriconazole and an echinocandin is recommended with marginal support in the first-line setting

### Management of Other Rare Infections

The inevitable depletion of CD4^+^ lymphocytes post allo-HSCT is associated with a risk of rare but highly lethal opportunistic infectious diseases including but not limited to *P. jirovecii* pneumonitis and cerebral and disseminated toxoplasmosis. Critical host factors suggested in adults, but also referring to children are a CD4^+^ lymphocyte count <200 cells/mm^3^ and the use of therapeutic doses to the equivalent of >0.3 mg/kg prednisone for >2 weeks (100, 101). Outcome depends on early recognition, immediate institution of appropriate therapy, appropriate supportive care and the reversal of immunosuppression.

*Pneumocystis* pneumonitis occurs almost exclusively in patients not taking the recommended standard prophylaxis trimethoprim/sulfamethoxazole (48, 50) and presents with respiratory distress that rapidly evolves into respiratory failure. Imaging findings are typically significant for symmetric intra-alveolar and interstitial infiltrates; the microbiological diagnosis is made by the detection of the organism in respiratory specimens (BAL or induced or expectorated sputum) by microscopy or nucleic acid amplification (100, 102). First-line antimicrobial therapy includes high-dose trimethoprim/sulfamethoxazole (equivalent to 15–20 mg/kg/day of trimethoprim), and supportive treatment consists of appropriate respiratory support, reduction of immunosuppression if feasible, and, in moderate and severe diseases (partial pressure of oxygen [PaO_2_] <70 mmHg on room air) and on a case-by-case basis, the adjunctive use of systemic corticosteroids (102, 103). Response to treatment is determined by the extent of pulmonary damage and may be expected after 5–7 days of treatment. Resistance to trimethoprim/sulfamethoxazole is not an issue, even in breakthrough infections, and a change of agent for reasons other than toxicity is generally not indicated. Of note, other concomitant opportunistic pulmonary infections, in particular CMV, need to be considered and excluded (102, 103).

Toxoplasmosis post allo-HSCT most frequently occurs as reactivation of a latent infection in a seropositive patient; primary infections may occur but are considered very rare. Most frequently, the CNS is affected, leading to diffuse encephalitis with variable onset and a diverse pattern of clinical symptoms; other manifestations include pulmonary or disseminated disease (104–107). Serological screening for toxoplasmosis prior to allo-HSCT is recommended for all patients, and screening by nucleic acid amplification by PCR of blood samples is advised in all seropositive patients for at least 6 months post HSCT (10, 39, 101). Diagnosis in a given patient is based on the evaluation of risk factors, clinical signs and symptoms and targeted imaging but ultimately requires the direct detection of parasites or their nucleic acids by PCR in blood, CSF, BAL or biopsies (101). Standard treatment includes pyrimethamine plus folinic acid (but not folic acid) in combination with either sulfadiazine or clindamycin for at least 6 weeks (10, 101). Whereas approximately 60% of patients may respond to treatment, neurological late effects may be expected in survivors and mandate careful evaluation of appropriate rehabilitation measures (101).

## SARS-CoV-2 Infections

The outbreak of severe acute respiratory syndrome coronavirus 2 (SARS-CoV-2) infections started in Wuhan, China, and the World Health Organisation classified it as a pandemic on 11 March 2020. The infection has had a major impact on paediatric haematology and oncology care, including HSCT. Over the recent months, national and international societies have made recommendations for the prevention and treatment of SARS-CoV-2 in the HSCT setting (108, 109). Most of the experience has been derived from adults and data in children are relatively scarce. Reports suggest that the majority of the children with cancer and post HSCT have either asymptomatic or mild SARS-CoV-2 infection (110) but severe disease courses with complications have also been described (111, 112). Two large registries reported on 11 and 19.9% of severe infections in paediatric cancer patients, and a mortality of 3 and 3.8%, respectively (112, 113). Overall, paediatric HSCT recipients seem to have similar risks of morbidity from SARS-CoV-2 as do healthy children (110). Interestingly, preliminary data reported by a Spanish group indicate that patients undergoing HSCT for immunodeficiencies have a higher risk of SARS-CoV-2 than general population, which may be due to the lack of development of thymus in these patients, which is associated with significant alteration of cellular immunity (114).

As in other infectious diseases such as influenza, prevention policies include the vaccination of household members and healthcare personnel (108, 115). All children undergoing HSCT, regardless of whether they have upper respiratory symptoms or not, should be tested for SARS-CoV-2, and test results should be negative prior to the start of conditioning. However, in case of a positive result, it is unclear whether it is always necessary to postpone HSCT and the final decision should be made on a case-by-case basis and according to the risk of cancer progression.

In addition, it has to be noted that access to the donor might be restricted by several factors, such as the infection of the donor by SARS-CoV-2 or logistical reasons such as travel restrictions across international borders due to the pandemic. Therefore, it is recommended to secure access to the stem cell product by freezing before the start of conditioning or to have an alternative donor as back-up (108).

Beside well-established supportive care strategies such as non-invasive ventilation and anti-coagulants, no specific treatment approach for HSCT recipients suffering from SARS-CoV-2 is evidence based and approved, in particular in the paediatric setting (108).

SARS-CoV-2 vaccination is now approved for children from the age of 12 years onwards in many countries but its value in paediatric HSCT recipients has yet to be evaluated. Studies in adult HSCT recipients have demonstrated that SARS-CoV-2 mRNA vaccines are well tolerated by HSCT recipients, that new GvHD developed in almost 10% of the patients after vaccination (116), and that 55% of the patients showed seroconversion after the first dose of the vaccine compared with 100% of controls (117). After the second dose, antibodies against SARS-CoV-2 were detectable in 82% of the patients (118). In children, however, data are lacking to date.

## Vaccination Policies

In children undergoing HSCT, transplant procedures as well as prevention and treatment of GvHD result in complex impairment of cellular and humoral immunity (60). It is well known that a significant proportion of HSCT recipients lose specific antibody titres against pathogens such as *Streptococcus pneumoniae, Haemophilus influenzae* type B and measles (119). The Infectious Diseases Society of America considers HSCT recipients as never vaccinated (119). During the first months after HSCT, most patients respond to vaccines to a lower extent than healthy individuals and so the timing of re-vaccination is a balance between the risk of immunisation failure if vaccination is given too early and risk of infection by vaccine-preventable diseases during the unprotected time period.

The PDWP of the EBMT recommends that re-vaccinations against diphtheria, tetanus, poliomyelitis, pertussis, hepatitis B, *Haemophilus influenzae* type B and pneumococci should be started at 6 months post HSCT in patients with leukocyte engraftment and a platelet count of ≥50,000/ μL irrespective of GvHD status and immune recovery and using the newborn diphtheria, tetanus, acellular pertussis (DTaP)/inactivated polio vaccine (IPV)/Hepatitis B virus (HBV)/*Haemophilus influenzae* type B (Hib) combination vaccine and the 13-valent pneumococcal conjugate (PCV13) vaccine (39). Notably, immunisation with non-live vaccines is safe during immunoglobulin replacement therapy as there is no specific risk besides non-response. Re-vaccination with live vaccines against measles, mumps, rubella and VZV should be started not earlier than 24 months post HSCT and should be given only to patients without GvHD, who ended immunosuppressive therapy ≥3 months ago, and who have ended immunoglobulin substitution (39).

## Summary

Allo-HSCT is an established treatment modality for paediatric patients with high-risk ALL. Infectious complications contribute significantly to patient morbidity and mortality after transplantation. However, over the decades, the manner in which HSCT is conducted has dramatically changed; this has had an impact on the type and timeline of infections in the post-transplant period. Not only transplant procedure but also recipient- and pathogen-specific factors may increase the risk of developing infectious complications after HSCT. Although the risk of bacterial, fungal or viral infections varies in different post-transplant phases, these infections can occur at any time until there is successful immunological reconstitution. Given the ongoing challenges in treating infectious complications after HSCT, research endeavours continue to evaluate novel diagnostic and therapeutic strategies. Moreover, continued investigation is necessary to help elucidate varying patterns of immune recovery after different methods of allo-HSCT. This may inform the development of an individualised approach to antimicrobial prophylaxis, empirical therapy and vaccination strategies in paediatric allo-HSCT recipients. A better understanding of the relationship between GvHD and infectious complications, as well as host–pathogen interactions, is required. All these efforts will result in improved graft selection, shortened neutropenia and enhanced immune reconstitution as well as the development of optimal prophylaxis and supportive care measures for all paediatric patients undergoing allo-HSCT for ALL.

## Data Availability Statement

The original contributions presented in the study are included in the article/supplementary material, further inquiries can be directed to the corresponding author.

## Author Contributions

All authors listed have made a substantial, direct, and intellectual contribution to the work and approved it for publication.

## Funding

This study received funding from the St. Anna Children's Cancer Research Institute, Vienna, Austria. The funders were not involved in the study design, collection, analysis, interpretation of data, the writing of this article, or the decision to submit it for publication.

## Conflict of Interest

The authors declare that the research was conducted in the absence of any commercial or financial relationships that could be construed as a potential conflict of interest.

## Publisher's Note

All claims expressed in this article are solely those of the authors and do not necessarily represent those of their affiliated organizations, or those of the publisher, the editors and the reviewers. Any product that may be evaluated in this article, or claim that may be made by its manufacturer, is not guaranteed or endorsed by the publisher.
